# A Pilot Randomised Control Trial of Digitally-Mediated Social Stories for Children on the Autism Spectrum

**DOI:** 10.1007/s10803-020-04490-8

**Published:** 2020-04-07

**Authors:** R. Hanrahan, E. Smith, H. Johnson, A. Constantin, M. Brosnan

**Affiliations:** 1 Centre for Applied Autism Research, Department of Psychology, University of Bath, Bath, BA2 7AY UK; 2 Present Address: University of Edinburgh, Edinburgh, EH8 9YL UK

**Keywords:** Autism, Social stories, Technology, RCT

## Abstract

Social stories is a widely used intervention for children on the autism spectrum, particularly within an educational context. To date, systematic reviews and meta analyses of the research evaluating social stories has produced mixed results, often due to a lack of methodological rigour and variability in the development and delivery of the social stories. To address the gap in methodological rigour, a pilot Randomised Control Trial (RCT) was conducted, incorporating a social stories intervention group (n = 9 children on the autism spectrum) and an attentional control group who received a poem (n = 6 children on the autism spectrum) using a digital platform to address variability. Digitally-mediated social stories were found to be effective in producing beneficial changes in behaviour outcomes, which were sustained at a six-week follow up.

## Introduction

Autism Spectrum Disorder (ASD) is characterised by persistent impairments in social interaction and communication across contexts, including deficits in social-emotional reciprocity, nonverbal communication, and relationship formation (APA 2013). Furthermore, restrictive and repetitive patterns of behaviour, activities and/or interests are displayed, including at least two of the following: repetitive motor movements, the insistence of sameness, fixated interests, and hyper or hypo sensitivity (APA 2013). Recent epidemiological studies have highlighted the increasing prevalence of ASD in young people (Manning-Courtney et al. 2013), with one in 59 children being diagnosed with ASD (Baio et al. 2018). Although not a diagnostic criterion, approximately two thirds of children on the autism spectrum^1^ exhibit maladaptive behaviours^2^ (or challenging behaviours, or problem behaviours; Hartley et al. 2008). These include internalising behaviours such as obsessions and withdrawal and/or externalising behaviours such as aggression and inattention (Hartley et al. 2008; see also Carter Leno et al. 2019).

Addressing maladaptive behaviours of children on the autism spectrum is of utmost importance as they negatively impact daily activities (Brereton et al. 2006). Maladaptive behaviours also impair the development of social skills, creating life-long barriers to inclusion (Rhodes 2014). In addition, maladaptive behaviours increase caregiver and family stress as they can be difficult to manage (Ludlow et al. 2012; O’Nions et al. 2018; Tomanik et al. 2004; Yacoub et al. 2018). Children on the autism spectrum may display maladaptive behaviours because of distress, confusion and frustration resulting from the inability to effectively communicate, understand social protocols and the misinterpretation of social cues (O’Connor 2009). Moreover, children on the autism spectrum’s high anxiety levels (e.g. van Steensel et al. 2011) may contribute to the presence of maladaptive behaviours, with higher levels of anxiety correlating with the increased presence of maladaptive behaviours in children on the autism spectrum (Cullain 2002; Rzepecka et al. 2011). The high prevalence of maladaptive behaviours in children on the autism spectrum and the association with caregiver stress and child anxiety emphasises the need for evidence-based interventions. Evidence-based interventions are needed to address the misunderstandings and confusion around social situations that may relate to these maladaptive behaviours.

Maladaptive behaviours within school settings take the form of self-injurious behaviour, aggressive/destructive behaviour and repetitive behaviour (Nicholls et al. 2019). Maladaptive behaviours are associated with an autism diagnosis and social stories are a promising intervention widely implemented in school-based settings for children on the autism spectrum (Gresham 2015; Kokina and Kern, 2010; Nicholls et al. 2019). Social stories are perceived by teachers of children on the autism spectrum to be an acceptable and effective intervention (100% and 95% respectively; Chan and O’Reilly 2008—a perception shared by parents: Whittingham et al. 2009) and are considered an evidence-based practice (NPDC 2014; NSP 2015). Social stories are simple, short, personalised narratives, composed of various sentence types which describe or coach an individual on a behaviour (Gray 2010). Social stories are written from the student’s perspective, suiting their cognitive ability, understanding, interests, and often capitalise on their visual learning strengths (Gray 2010). Thus, many social stories incorporate picture symbols, cartoons or photographs alongside text. Social stories are applicable to a variety of maladaptive behaviours (Reynhout and Carter 2007) with the principle aim of objectively informing individuals of important social information surrounding these behaviours. This is achieved by explicitly detailing social cues, perspectives and responses (Gray 2010). In addition to explicit sentences that describe the situation, coaching sentences detail appropriate behaviour in that situation, such as an appropriate alternative behaviour to a maladaptive behaviour. Thus, their central premise is to provide a medium for learning social information to better an individual on the autism spectrum’s understanding (Gray 2010). As a consequence of this increased understanding, or a decrease in anxiety (see above), there is potential to address maladaptive behaviours, although the precise mechanism by which social stories work is unknown (Kokina and Kern 2010).

As examples, following the introduction of social story interventions, chair tipping, shouting and inappropriate staring significantly decreased for three students on the autism spectrum (Scattone et al. 2002). For one student chair tipping significantly reduced from 50% during baseline to 4.6% during the intervention period (Scattone et al. 2002). Moreover, the ‘talk outs’ of an eight-year-old boy on the autism spectrum, defined as talking without raising one’s hand, significantly dropped from an average of 11.2 per 30-min during baseline to 2.3 per 30-min during the intervention (Crozier and Tincani 2005). These are examples of reducing inappropriate behaviour. Also, social stories can address maladaptive behaviours by increasing appropriate behaviours, such as suitable play skills (Barry and Burlew 2004) or lunchtime eating behaviour (Bledsoe, Smith and Simpson 2003). For example, mouth wiping increased from a median frequency of zero at baseline to one during the intervention (Bledsoe et al. 2003). However, these studies either adopted a case study approach or had a very small sample size (Kuoch and Mirenda 2003; Reynhout and Carter 2006), limiting the generalisability of these findings to the maladaptive behaviours of other children on the autism spectrum. In addition, behaviour often returns to baseline after the intervention and any long-term effects are questionable. These children also served as their own controls reducing the ability to reliably attribute the behaviour outcomes to the social story intervention (Sansosti et al. 2004), as a change in behaviour may have been due to regular and supportive one-to-one attention (Rhodes 2014).

Despite there being a large number of case studies evidencing the positive effects of social stories upon maladaptive behaviours of children on the autism spectrum, systematic analyses and meta analyses highlight major inconsistencies within the literature (Reynhout and Carter, 2006; Quirmbach et al. 2009; Kokina and Kern 2010; Test et al. 2011; Kokina and Kaczmarek 2014; Sani et al. 2014; Wright et al. 2016; Qi et al. 2018). Typically, only a small number of studies meet inclusion criteria (e.g. 6: Karkhaneh et al. 2010), with variable effectiveness (e.g. 51% of included studies: Kokina and Kern 2010) and effect sizes (e.g. small to large: McGill et al. 2015). A recent analysis of 16 literature reviews and meta-analyses identified 55 studies for inclusion and concluded that they do not support the use of social stories to improve social skills or behaviour (Garwood and Van Loan 2019). It may be the case that social stories are more effective at reducing inappropriate behaviours specifically (Kokina and Kern 2010; Qi et al. 2018). These analyses consistently call for better controlled studies and suggest that inconsistencies in effectiveness are attributable to a lack of fidelity (e.g. McGill et al. 2015; Test et al. 2011), and consequently social stories should not be considered an evidence-based practice (Bozkurt and Vuran 2014; Horner et al. 2005; Qi et al. 2018).

Randomised Control Trials (RCTs) allow for cause and effect between the intervention and behaviour change to be established, increasing methodological rigour for autism interventions (Mesibov and Shea 2011). However, to date, only four between group RCTs have been conducted using social stories, all of which did not meet inclusion criteria for a review by Wright et al. (2016). The studies generally failed to successfully follow Gray’s social story criteria and interventions tended to lack an individualised story constructed for the specific needs of the child and were vulnerable to selection and reporting bias (Marshall et al. 2016; Wright et al. 2016). In addition, social stories were read for a single day in all four studies. Consequently, Marshall et al. (2016) argue that there is ‘a strong justification to conduct a well-designed, ecologically valid, large-scale RCT on the effectiveness of Social Stories which used individualised stories within a school setting’ (p. 2). Marshall et al. (2016) conducted a feasibility study for RCTs on the use of social stories for children on the autism spectrum. The authors concluded that teachers are most appropriate to complete the outcome measures, which should include a measure of how close the child is to the goal of the social story, and that a 6-week follow-up was an appropriate time scale.

One way to overcome research inconsistencies is to use digital technology within RCTs (Goldsmith and LeBlanc 2004), and social stories for children on the autism spectrum may be particularly amenable to delivery through digital technology (e.g. Kennedy et al. 2019; Ghanouni et al. 2019). Digital storytelling is a combination of traditional oral storytelling supported by personalised digital images (e.g. of the child), graphics and sound, presented on a computer (Lambert 2013; Robin 2006; 2008). This develops social narratives that support learning for children on the autism spectrum (Doody 2015; Hale and Schmidt, 2018; Harjusola-Webb et al. 2012). Digitally-mediated social stories combine digital storytelling with social narratives structured according to Gray’s criteria (Ying et al. 2016). There are benefits of digitally-mediated social stories for those who have difficulties with social interaction, enabling greater intensity of interaction with the content of the story. Computers can provide a more consistent and structured environment for the story, enabling repetition and direct feedback, and can offer the child more control over the learning experience. Digital technology can also enhance visual support, self-monitoring, and rewards, all of which can be personalised to the child (Constantin et al. 2017; Moore 2008; Odom et al. 2003; Ozdemir 2010; Segers and Verhoeven, 2005; Smith et al. 2020; Yildirim et al. 2001). Digital technology is particularly beneficial when it is embedded in the classroom (Sutherland et al. 2004). This is pertinent as a recent systematic review has highlighted that school-based interventions for autism are resource intensive and usually delivered by researchers away from the classroom. This highlights the need for studies documenting effective interventions that are feasible in school settings (Anderson et al. 2018; Smith et al. 2020; Sutton et al. 2019). Whilst digital interventions have great promise within school settings, evidence for best practice is yet to be established (Zervogianni et al. 2020a, b). Digital interfaces, such as tablets, are relatively inexpensive and readily available within many classrooms, with the potential to reduce variability and enhance fidelity in a user-friendly manner (Kagohara et al. 2013; see Almutlaq and Martella 2018; Cazaux et al. 2019; Lorah 2018; Muharib et al. 2019). Children on the autism spectrum can show a preference for interventions being delivered through tablet devices (such as iPads), compared to traditional methods (Bouck et al. 2014; Mancil et al. 2009; for systematic reviews of the benefits of iPads for autism interventions see Alzrayer et al. 2014; Kagohara et al. 2013).

The present study therefore piloted a digitally-mediated social stories RCT intervention for children on the autism spectrum for the first time in a school setting. We hypothesised that the behaviour targeted for intervention would reduce in both frequency and intensity, and that it would be closer to the desired goal of the social story in the intervention group compared to the control group. We also hypothesised that there would be an increase in understanding and a reduction in anxiety in the intervention group compared to the control group.

## Method

### Participants

Fifteen pupils (14 male and 1 female) aged 4–10 years (*M* = 6.8, *SD* = 2.15) took part in the study. All pupils attended a special educational needs (SEN) school in the South West of England and had a formal diagnosis of ASD from a clinician using established international criteria (World Health Organization 2018), with no co-occurring conditions diagnosed. Fourteen participants were White British and one was British Asian, and specific data on social economic status was not recorded. Children who were minimally verbal and/or those without a functional understanding of English; along with children who had received a social story within the past 6 months; or who were receiving another intervention at the time of this study were excluded.

Participants were recruited via opportunity sampling. The researcher (first author) worked within the school as a teaching assistant for class teachers. The researcher met with class teachers to identify children on the autism spectrum who did not meet any of the exclusionary criteria and, who had a behaviour suitable for a social story intervention. Once identified, parental consent was obtained as was assent from the child. Participants’ teachers confirmed that they would not implement any additional interventions for these children during the research period. Full ethics approval from the University of Bath Research Ethics Committee was obtained for this study.

### Design

The pilot study adopted a quasi-experimental RCT between-subjects design, in order to overcome previously flawed social story research (Marshall et al. 2016; Wright et al. 2016). This RCT examined the impact of a digital social story intervention through comparison with an attentional control group, who received a simple poem of comparable length called ‘Witch, Witch’. This ensured that all the children had the same amount of time on the iPad and one-to-one time with the Researcher. Control participants were simply read the poem using the iPad, there was no stated objective, such as increasing social or listening skills. Participants were randomly assigned, overcoming any risk of selection bias. The social stories and control poem were administered over a two-week period, as social story interventions lasting less than three weeks produce the largest treatment effects (McGill et al. 2015). Thus, two-weeks is an appropriate intervention length (Kokina and Kern 2010).

For each participant in the intervention group, the Researcher developed a unique social story to address the target behaviour identified by the class teacher. The meeting with the teacher identified the maladaptive behaviour to be targeted in terms of positive goals (this could be to increase an appropriate target behaviour or reduce an inappropriate target behaviour). Examples of goals used included: to take turns when using the bikes in the playground; to sit appropriately whilst on the carpet; to learn about personal space and give my friends and teacher their own personal space (see supplementary material for full details of story texts). A brief guide, devised by the Educational Psychologist (second author), was used to help elicit potential antecedents, consequences and communicative function relating to the maladaptive behaviour. Information was also gathered from the class teacher so that the stories could be tailored for the abilities and interests of each child. Training and support with story writing was provided by the Educational Psychologist, who also checked to ensure they met with Carol Gay’s criteria (see appendix for further details). This ensured the story was structured appropriately, ‘Wh’ questions were answered, sentence types were appropriate and balanced and wording was correct (e.g. literal, avoiding ‘must’). Stories were presented on an iPad and included pictures and/or photos that were personalised for each child. The Researcher read the social story together with the child (one-to-one, without the teacher being present) once a day for two weeks (ten consecutive school days). The social stories were introduced in a positive manner, in a suitable setting within the school for each child.

Following random allocation to one of the two conditions, using an online random number generator, there were nine participants in the intervention group and six in the control group. In order to reduce observer bias, each participant’s class teacher was blind to which condition the participant was in (Torgerson & Torgerson 2001). The class teacher was asked to rate the participating child on the measures below (after Marshall et al. 2016)—these ratings being blind to the Researcher. Questionnaires were completed daily and weekly (see below) at three time-points: a) during the baseline week; b) throughout the intervention phase (2 weeks); c) at the 6-week follow-up. Social stories were developed to be administered to the control group after the 10-week follow up to ensure every participant had access to the intervention regardless of their treatment allocation—no data was collected for this.

### Measures

Previous research has often drawn conclusions on the effectiveness of social story interventions by measuring behaviour frequency (Rhodes 2014), however, intensity of the behaviour is also a critical dimension of maladaptive behaviours (Goodley 2001; Sofronoff et al. 2004; Haggerty et al. 2005). Therefore, a measure of intensity was included in the present study as was the measure of closeness to the social story goal, identified by Marshall et al. (2016). However, by purely assessing behaviour outcomes an increase in understanding of behaviour cannot be identified, despite understanding being argued to be the central premise of social story interventions (Gray 2010) with some evidence from the neurotypical literature indicating that social stories increase a child’s understanding of the social world (Toplis and Hadwin 2006). In addition, it has been speculated that social stories may reduce the behaviour-related anxiety of children on the autism spectrum (Cullain 2002; Rzepecka et al. 2011; O’Connor 2009). Therefore, perceived understanding and anxiety in the child was also assessed in the present study.

#### Daily Behaviour Diary

At the end of each school day, the class teacher rated how close the participant was to reaching their pre-specified social story goal using an 11 point Likert response scale of 0 (not met goal) to 10 (goal completely met). This measure was taken from Marshall et al.’s feasibility study (2016) as it was found to be the most reliably completed assessment by teachers (compared to other measures such as the Social Responsiveness Scale-2 or the Strengths and Difficulties Questionnaire). The main purpose of the daily behaviour diary was to ensure the teacher stayed focused upon the target behaviour of the child, and as a check to ensure that daily rating matched weekly ratings.

#### Weekly Teacher Questionnaire

At the end of each school week, the teachers were asked to rate the participant on the 11-point Likert scale described above for closeness to the social story goal (Marshall et al. 2016). Four variables were also rated: behavioural frequency of the identified behaviour, behavioural intensity of the identified behaviour, perceived child’s understanding of behaviour and perceived behaviour-related anxiety of the child. For example, ‘Please rate on a scale of 0 to 10 the child’s level of understanding of the behaviour’. This questionnaire was completed at the end of each week throughout a four-week period. Following an ABA design, week one was baseline (no intervention), followed by the intervention in weeks two and three and the removal of the intervention for week four. The questionnaire was administered again after an additional six weeks without intervention as a follow-up (i.e. 10 weeks after the start of the intervention).

#### iPad and App

The individualised social stories were written for the intervention group by the Researcher using a prototype of the social stories app ‘Stories Online For Autism’ (SOFA-app.org, from Autumn (Fall), 2020). SOFA-app was co-developed with the autism community and is freely available for IOS and Android. During weeks two and three, the intervention group were read their social story every day and the control group were read the control poem every day, each lasting approximately five minutes. All were read on an iPad by the Researcher in a quiet area of the participant’s classroom with minimal distractions present. This familiar person and environment ensured that all participants were comfortable when being read to.

### Data Analysis

Following data collection, all data were input into SPSS. For the intervention group whose social stories increased appropriate behaviours, the scoring scales for the frequency and intensity measures were reversed. This was to ensure that they matched the social stories which decreased inappropriate behaviours, such that a decrease in frequency and intensity is consistently viewed as the beneficial outcome. A Shapiro–Wilk normality test identified the weekly questionnaire data to be non-normally distributed (*p* < 0.05). Therefore, this data was deemed appropriate for non-parametric statistical tests.

A correlation was conducted to assess the relationship between the mean scores on the daily goal-based measure and the weekly goal-based measure, in order to highlight the reliability of the weekly measure. As the weekly data was non-parametric, a Spearmen’s rank order correlation was conducted on the data from week one, two, three and four. All correlations were significant (*p* < 0.05), hence the weekly goal-based measures were significantly correlated with the mean of the daily goal-based measures each week.

## Results

Results are arranged by analysis of each weekly questionnaire measure. Firstly, line graphs display the mean scores on each measure for both the intervention and control group across week one (baseline), two (intervention), three (intervention), four (post-intervention) and the six-week follow up. Then, Wilcoxon Signed-Ranks tests were conducted to test for differences between week one and the six-week follow up on each measure for the intervention and control group. Following this, Mann–Whitney U tests were conducted to test for differences between the intervention and control group on each measure at each time point. N = 15 in all statistical tests. Finally, effect sizes (Cohen’s d, Cohen, 1992) were calculated for the intervention after 4 weeks and the 6-week follow up between the intervention and control groups. In addition, the within group effect sizes were calculated^3^ for the intervention group comparing week 1 with week 4 then week 1 with week 6 follow up.

**Fig. 1:**
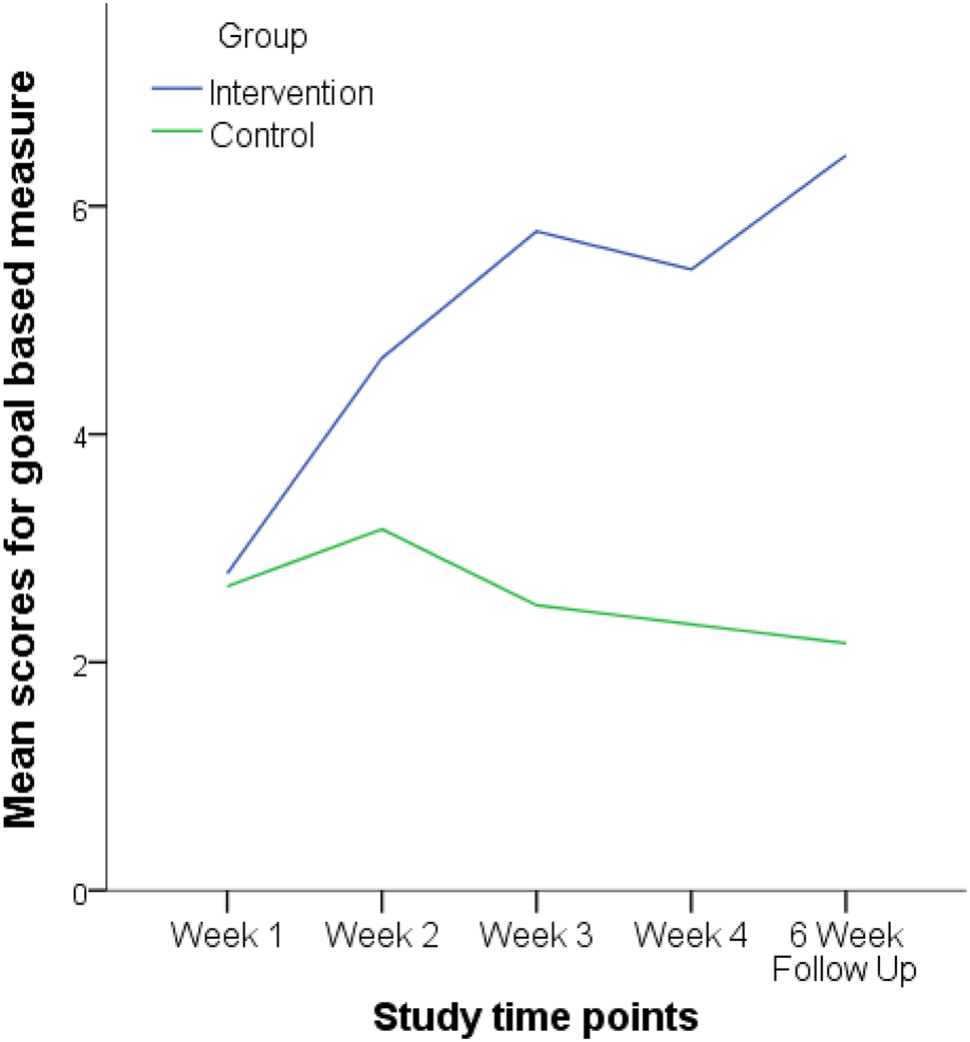
Line graph showing how close the intervention and control group were to achieving their social story goal (0 = goal not met; 10 = goal completely met)

The weekly goal-based measure showed how close the participants were to achieving their social story goal (see Fig. 1*)*. A Wilcoxon Signed-Ranks test indicated that there was a significant difference between week one (baseline) and the six-week follow up on the goal-based measure for the intervention group (*Z* = 2.530, *p* = 0.011), but not the control group (*Z* = 0.535, *p* = 0.593). This indicates that the improvements in the goal-based measure had been maintained for the intervention group*.*

A Mann–Whitney U test indicated there was no significant difference between the intervention and control group on the goal-based measure during week one (baseline) (*U* = 27, *Z* = 0.001 *p* = 1.000) and week two (first week of intervention) (U = 16.5, Z = 1.267, *p* = 0.224). However, the intervention group (*Mdn* = 6, *range* = [2–8]) scored significantly higher than the control group (*Mdn* = 2.5, *range* = [1–3]) on the goal-based measure during week three (*U* = 4.5*, Z* = 2.695, *p* = 0.005*)* and the intervention group (*Mdn* = 6, *range* = [2–8]) scored significantly higher than the control group (*Mdn* = 2, *range* = [2–3]) in week four (*U* = 8, *Z* = 2.319, *p* = 0.026). Also, the intervention group (*Mdn* = 7, *range* = [3–9]) scored significantly higher than the control group (*Mdn* = 2.5, *range* = [1–3]) in the six-week follow up (*U* = 3, *Z* = 2.894*, p* = 0.003). The between group effect sizes for the 4-week and 6-week follow up were d = 1.83 and d = 2.20, respectively. The within group effect sizes for the intervention group were d = 1.78 comparing week 1 to week 4, and d = 1.51 comparing week 1 to week 6 follow up.

**Fig. 2:**
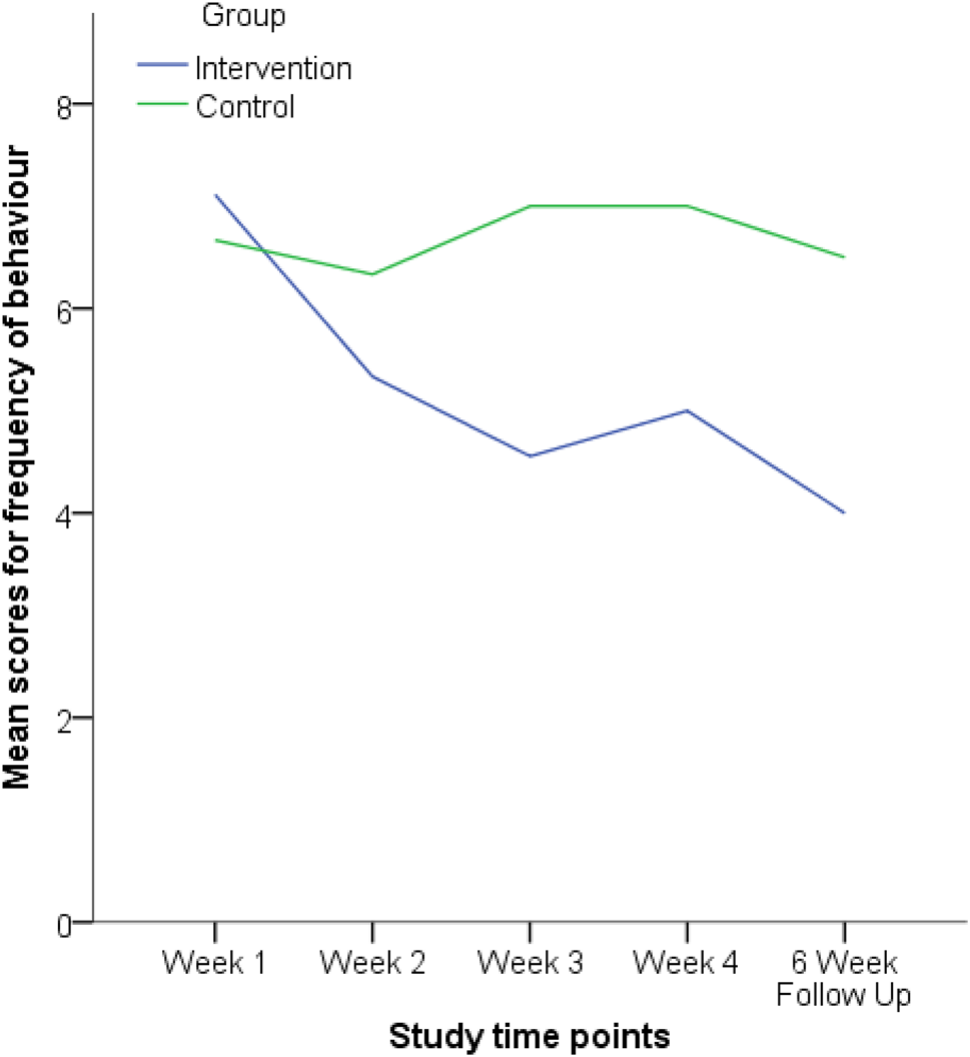
Line graph showing the intervention and control groups frequency of behaviour across the study

The weekly frequency measure showed how frequently the participants’ target behaviour occurred (see Fig. 2). A Wilcoxon Signed-Ranks test indicated that there was a significant difference in frequency between week one (baseline) and the six-week follow up for the intervention group (*Z* = 2.410, *p* = 0.016), but not for the control group (*Z* = 0.333, *p* = 0.739). This indicates that the reduction in behaviour frequency had been maintained for the intervention group*.*

Mann–Whitney U tests indicated that there was no significant difference between the intervention and control group on the frequency measure in week one (*U* = 22, *Z* = 0.605, *p* = 0.607), or week two (*U* = 15.5, *Z* =  − 1.387, *p* = 0.181). However, the intervention group (*Mdn* = 4, *range* = [3–8]) scored significantly lower than the control group (*Mdn* = 7, *range* = [6–8]) on the frequency measure in week three (*U* = 6, *Z* = 2.516, *p* = 0.012) but this difference was not significant in week four (U = 14, Z = 1.547,* p* = 0.145). Also, the intervention group (*Mdn* = 4, *range* = [2–6]) scored significantly lower than the control group (*Mdn* = 6.5, *range* = [5–8]) at the six-week follow up (*U* = 7, *Z* =  − 2.394*, p* = 0.018). The effect sizes for the 4-week and 6-week follow up were d = 1.09 and d = 1.69, respectively. The within group effect sizes for the intervention group were d = 0.53 comparing week 1 to week 4, and d = 1.09 comparing week 1 to week 6 follow up.

**Fig. 3:**
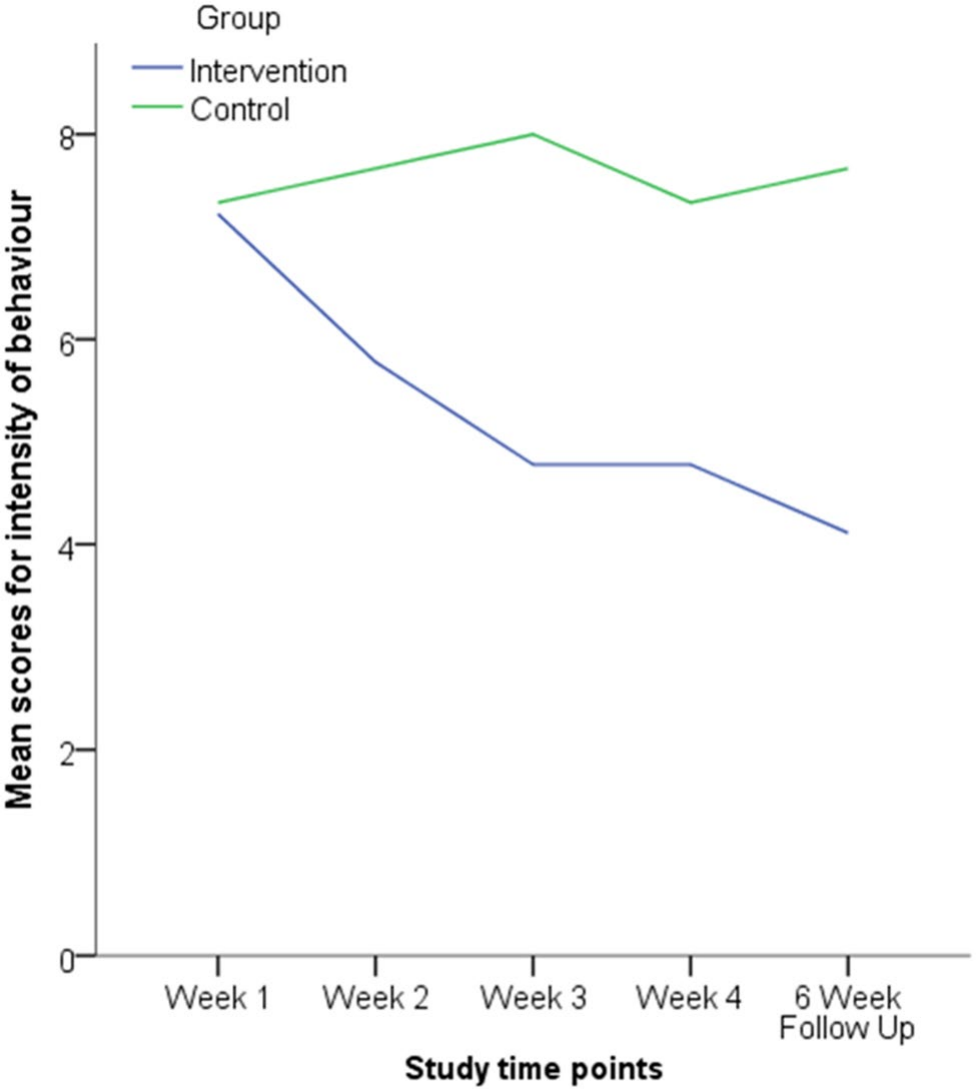
Line graph showing the intervention and control groups intensity of behaviour across the study

The weekly intensity measure showed how intense participants’ target behaviour had been (see Fig. 3). A Wilcoxon Signed-Ranks test indicated that there was a significant difference in the intensity measure between week one (baseline) and the six-week follow up for the intervention group (*Z* = 2.437, *p* = 0.015), but not the control group (*Z* = 0.707, *p* = 0.480). This indicates that the reduction in behaviour intensity had been maintained for the intervention group*.*

Mann–Whitney U tests indicated that there was no significant difference between the intervention and control group on the behaviour intensity measure in week one (U = 26, Z = 0.123, p = 0.955) and week two (U = 12, Z = 1.799, p = 0.088). However, the intervention group (*Mdn* = 4, range = [1–9]) scored significantly lower than the control group (*Mdn* = 8, *range* = [7–9]) on the intensity measure during week three (*U* = 7.5, *Z* =  − 2.332, *p* = 0.018). The intervention group (*Mdn* = 4, *range* = [2–9]) also scored significantly lower than the control group (*Mdn* = 7.5, *range* = [6–8]) in week four (*U* = 7, *Z* =  − 2.385, *p* = 0.018). Furthermore, the intervention group (*Mdn* = 4, *range* = [1–7]) scored significantly lower than the control group (*Mdn* = 7.5, *range* = [6–9]) at the six-week follow up (*U* = 4.5, *Z* = 2.685, *p* = 0.005). The effect sizes for the 4-week and 6-week follow up were d = 1.63 and d = 2.04, respectively. The within group effect sizes for the intervention group were d = 0.68 comparing week 1 to week 4, and d = 1.03 comparing week 1 to week 6 follow up.

**Fig. 4:**
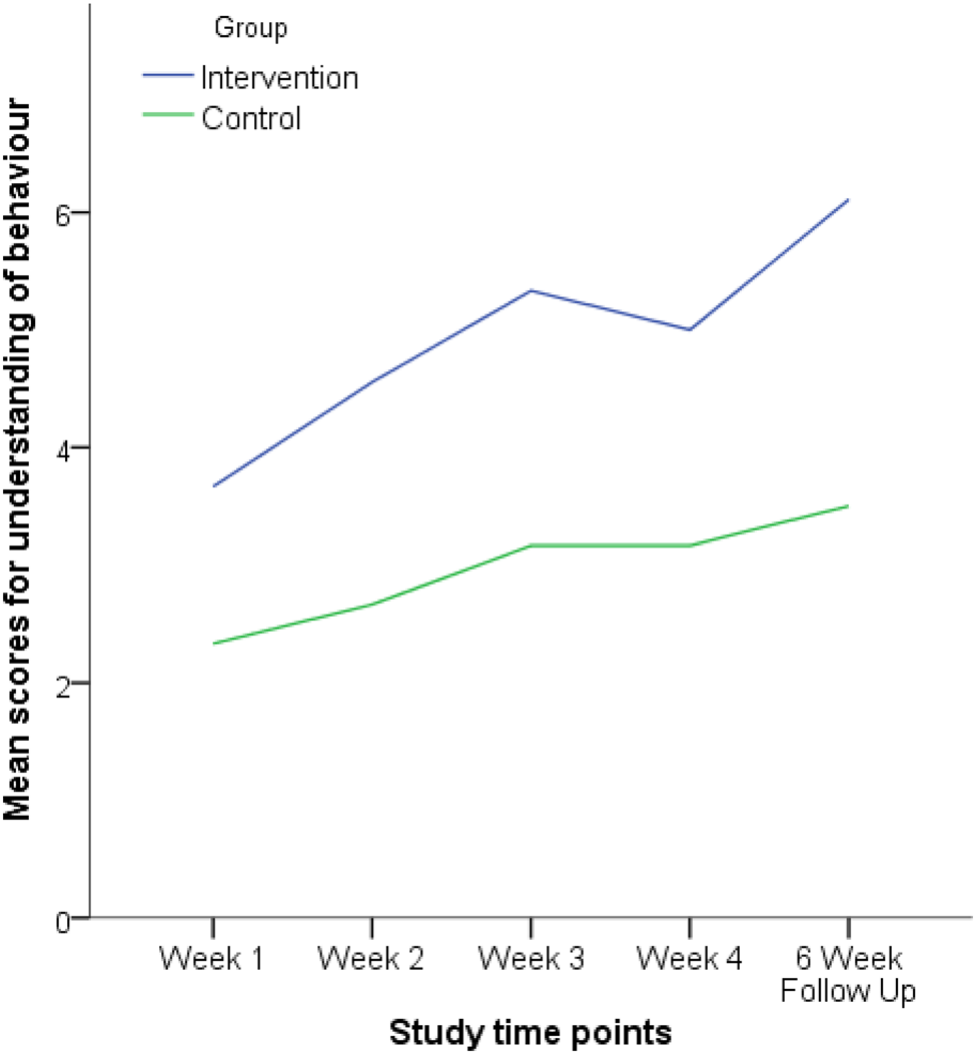
Line graph showing the intervention and control groups understanding of behaviour across the study

The weekly understanding measure showed participants’ level of understanding about their target behaviour (see Fig. 4). A Wilcoxon Signed-Ranks test indicated that there was a significant difference in the understanding of behaviour between week one (baseline) and the six-week follow up for the intervention group (*Z* = 1.973, *p* = 0.049), but not the control group (*Z* = 0.633, *p* = 0.102). This indicates that the improvements in understanding had been maintained for the intervention group*.*

A Mann–Whitney U test indicated that there was no significant difference between the intervention and control group in understanding during week one (*U* = 11.5, Z = 1.897, *p* = 0.066). However, the intervention group (*Mdn* = 5, *range* = [3–7]) scored significantly higher on the measure of understanding compared to the control group (*Mdn* = 2, *range* = [2–5]) in week two (*U* = 7, *Z* = 2.423, *p* = 0.018). The intervention group (*Mdn* = 5, *range* = [4–8]) scored significantly higher than the control group (*Mdn* = 3, *range* = [2–6]) in week three (*U* = 7, *Z* = 2.400, *p* = 0.018). Also, the intervention group (*Mdn* = 5, *range* = [1–8]) scored significantly higher than the control group (*Mdn* = 3, *range* = [1–5]) in week four (*U* = 9, *Z* = 2.174, *p* = 0.036) but this difference was not significant at the six-week follow up (U = 11.5, Z = 1.840, *p* = 0.066). The effect sizes for the 4-week and 6-week follow up were d = 1.13 and d = 1.19, respectively. The within group effect sizes for the intervention group were d = 0.69 comparing week 1 to week 4, and d = 0.89 comparing week 1 to week 6 follow up.

**Fig. 5:**
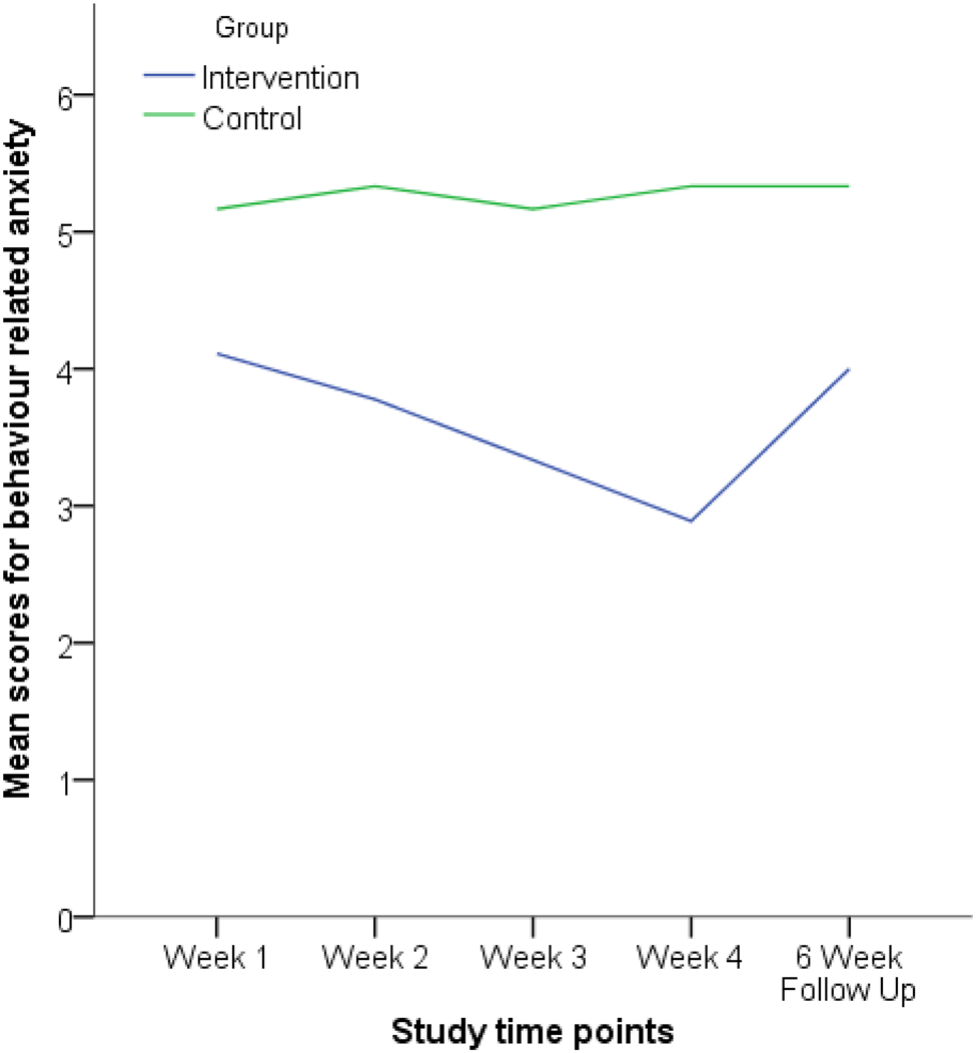
Line graph showing the intervention and control groups behaviour-related anxiety across the study

The weekly measure of anxiety showed participants’ level of anxiety regarding their target behaviour (see Fig. 5). A Wilcoxon Signed-Ranks test indicated that there was no significant difference in behaviour-related anxiety between week one (baseline) and the six-week follow up for the intervention group (*Z* = 0.001, *p* = 1.00) or the control group (*Z* =  − 0.577, *p* = 0.564). This indicates that the reduction in behaviour-related anxiety had not been maintained for the intervention group*.*

Mann–Whitney U tests indicated there was no significant difference between the intervention and control group on the anxiety measure in week one (*U* = 18, *Z* = 1.089, *p* = 0.328), week two (*U* = 14, *Z* = 1.578, *p* = 0.145), or week three (*U* = 11, *Z* = 1.924, *p* = 0.066). However, the the intervention group (Mdn = 2, range = [2–5]) scored significantly lower than the control group (Mdn = 5, range = [3–8]) on anxiety in week four (U = 6.5, Z = 2.481, p = 0.012) but this difference was not significant at the six-week follow up (*U* = 19, *Z* = 0.954, *p* = 0.388). The effect sizes for the 4-week and 6-week follow up were d = 1.57 and d = 0.61, respectively. The within group effect sizes for the intervention group were d = 0.51 comparing week 1 to week 4, and d = 0.04 comparing week 1 to week 6 follow up.

Finally, although numbers were small, an exploratory visual analysis was made of the social stories categorised by the Researcher as decreasing inappropriate behaviour (n = 4) compared to the social stories which aimed to increase appropriate behaviours (n = 5). On the measures described above, it appeared that increasing appropriate behaviours was rated more positively than decreasing inappropriate behaviours, especially for the measures of understanding and behavioural intensity.

## Discussion

Despite social stories being a widely used intervention for children on the autism spectrum, only four previous RCTs have been identified, all of which targeted a single behaviour for all participants (three for social skills related to playing a game, one for learning emotions). These studies were all were delivered over a single day, resulting in exclusion from meta analyses (Marshall et al. 2016; Wright et al. 2016). In addition, none adhered to Carol Gray’s guidelines regarding story development or delivery. In order to address this limitation in the literature, for the first time the present study utilised a rigorous, ecologically valid pilot RCT to investigate the effectiveness of a social story intervention in addressing maladaptive behaviours in children on the autism spectrum within an educational context using an iPad. The changes in maladaptive behaviours can be brought about by either reducing inappropriate behaviour (such as talking out in class) or increasing appropriate behaviour (such as wiping food from mouth; Bledsoe et al. 2003; Kokina and Kern 2010; Scattone et al. 2002). To minimise variability in the development and delivery of the social stories, the intervention was administered through an iPad-based app specifically co-developed with the autism community for this purpose.

Overall, the findings were positive, identifying significant improvements between the week one baseline and the follow up ten weeks later (six weeks after the end of the intervention). Large effects were found for the goal-based outcome, behaviour intensity, behaviour frequency and perceived behaviour understanding measures for the intervention group, but not the control group. However, no significant difference was identified for the intervention group between week one and the follow up on the perceived behaviour-related anxiety measure. Importantly, no significant differences were identified between the intervention and control group on all measures during week one (baseline), indicating the two groups were comparable prior to the intervention. In addition, whilst the trends were in the predicted direction, after one week of intervention there were no significant differences between the intervention and control groups, except on the understanding measure. However, significant differences emerged between the two groups after the second week of intervention (week three) for all measures (except anxiety), suggesting that two weeks may be a useful guide when considering the length of the intervention in future when social stories are read once a day (see also McGill et al.^2015^). Also, significant differences between the intervention and control group were evident at the six-week follow up on the goal-based, frequency and intensity measures, but not the understanding and anxiety measures. The between group effect sizes and the within group effect sizes for the intervention group (comparing week 1 with week 4 and week 6 follow up) were all medium to large effect sizes, again with the exception of anxiety at the 6 week follow up.

In a recent systematic review of the effects of social stories on individuals on the autism spectrum, Qi et al. (2018) highlighted that participant numbers ranged from 1 to 6 (with an average of 2.5) in the studies they reviewed. Whilst the numbers in the present study are larger than this, they are still small and considered a pilot study, which needs to be borne in mind when considering the results. Non-parametric data also limited the analyses that could be undertaken. The fact that significant differences could be identified with small numbers may be useful for future research, especially given the call for large-scale studies by Marshall et al. (2016). Whilst large-scale studies are to be welcomed, Marshall et al. recruited 50 participants through 39 schools. Their analysis suggests 180 participants would be ideal for a between group RCT, such as this one in the present study. Extrapolating from their figures, this would necessitate the involvement of 133 schools, to assess 90 children receiving social stories (and 90 children in a control group). The present study suggests that significant findings can be identified with 10% of this number, however it is important to note that many of the findings would not retain significance if statistical adjustments were made for multiple testing, which is a limitation of the present study. In spite of this limitation, the findings were in line with expectation and it may be that the digital technology serves to reduce variability in the development and delivery of social stories, which in turn enhances effectiveness. It may also be the case that the SEN setting of the present study, or the profile of the children on the autism spectrum attending SEN schools (compared to mainstream schools) impacts upon these findings (Herrera et al. under review). Whilst no co-occurring conditions were diagnosed within the present sample, it is likely that children on the autism spectrum attending SEN provision may be achieving at an academically lower level than children on the autism spectrum in mainstream provision. There is a small amount of evidence that lower cognitive ability relates to greater social story effectiveness in children on the autism spectrum (Kokina and Kern 2010). A limitation of the study is that it was not possible to further characterise the intervention and control groups in terms of their diagnoses, cognitive and language functioning, and special education eligibility and services. This was a result of a lack of access to existing records which can be addressed in future research.

These promising findings may be due to the social stories being written in a standardised and individualised way, in line with Gray’s guidelines (Gray 2010). This maximises intervention fidelity and may explain the maintained changes in maladaptive behaviours which previous research has failed to identify (Test et al. 2011). Despite this, replication of these findings and the assessment of behaviour outcomes beyond six-weeks would be of further benefit in assessing the effectiveness of social stories in changing children on the autism spectrum’s maladaptive behaviours. Future research can explore the extent to which the structure and support within the SOFA-app, combined with a consistent method of delivery, enables non-experts to develop and deliver stories consistent with Gray’s guidelines. Also, of interest to future research is the way that such an app could enable extended application of a social story as and when it is needed over a period of months, or even years. Whether the social stories are written by a parent/carer or an expert practitioner, if such an app enables a parent/carer to consistently deliver an intervention at the point it is needed, this opens up a potentially fruitful avenue for future research and practice.

Visual inspection of the figures above suggests a fairly linear effect across the two weeks of intervention, and it is not known what would happen if the intervention were continued for future weeks. Overall the intervention group were rated around 7 out of 10 for positive aspects (goal/understanding) and 4 out of 10 for negative aspects (intensity/frequency/ anxiety), suggesting that further improvements could still be gained. These measures are all teacher-based assessments and the teacher has been identified as the most reliable informant by Marshall et al. (2016) who also recommend the use of the goal-based measure. The present study is consistent with Marshall et al. in finding this measure useful, although it must be acknowledged that all our measures are based upon the perception of the class teacher. In addition, whilst the class teacher was formally blind to the condition of each child, changes in the child’s behaviour may have impacted upon the blinding process.

The potential explanatory mechanisms for social story efficacy were also explored. A significant difference between week one and the six-week follow up in perceived understanding of behaviour was evident for the intervention but not the control group. This is consistent with the proposal that social stories improve children on the autism spectrum’s understanding (Gray, 2010), which may have accounted for behaviour improvements in frequency, intensity and closeness to social story goal. Thus, elucidating a theoretical rationale for social story interventions (Murphy et al. 2005) and potentially providing support for an account of impaired Theory of Mind and perspective-taking in children on the autism spectrum (Baron-Cohen et al. 1985), which reduces social understanding and drives maladaptive behaviours (O’Connor 2009). However, a non-significant difference in understanding was identified between the intervention and control group at the six-week follow up, despite the intervention group scoring significantly higher than the control group during week two, three and four, and a clear visual increase in understanding between week four and the six-week follow up being present (see Fig. 4). Thus, until this research has been replicated with a larger sample to assess if the six-week follow up data reaches statistical significance, caution needs to be taken in positing increased behaviour-related understanding as a definitive explanatory mechanism. Also, the present study did not collect any direct data from the children on their understanding of behaviour, which can be addressed in future research.

Previous literature has suggested that social stories are effective in reducing anxiety, which consequently results in behaviour improvements (Cullain 2002; O’Connor 2009). Whilst there was some evidence consist with this in the present study, this only seems to be the case whilst the intervention is running. It may be, therefore, that continuing the intervention beyond two weeks (see above) may be particularly beneficial for reducing anxiety related to maladaptive behaviours. Social stories are used to prepare children on the autism spectrum for an upcoming event (such as going to the dentist: Kokina and Kern 2010), and it may be that anxiety is more relevant for this type of social story goal, compared with addressing maladaptive behaviours. A limitation of the present study is that only the teachers’ perceptions of anxiety levels were obtained and future research can incorporate additional rigorous and independent assessments of anxiety. We have used the term maladaptive behaviours in preference to challenging or problematic behaviours. Whilst these latter terms raise issues concerning ‘challenging/ problematic for whom?’, the term maladaptive also has connotations, and whether the absence of a socially normed behaviour (such as wiping food from mouth) is maladaptive is open to debate. Previous research has suggested that social stories may be more effective in reducing inappropriate maladaptive behaviour compared with increasing appropriate behaviours (Kokina and Kern 2010; Qi et al. 2018). Whilst numbers in the present study were too small to analyse this formally, a visual analysis suggested that, if anything, increasing appropriate behaviours was more effective than reducing inappropriate behaviours. Whilst speculative, it may be that the engaging and appealing features of visual media and a touch screen digital device increases the motivation and interest of children on the autism spectrum leading to promising gains in self-directed learning, independence, and pro-social outcomes (Ghanouni et al. 2019; Hong et al. 2017; Kim et al. 2014; Vandermeer et al. 2015). As an example, the SOFA-app enables photos taken by the digital device (tablet or smartphone) to illustrate the stories. Children on the autism spectrum can be strong visual learners and highly motivated by viewing images of the self on a computer screen (Wert and Neisworth 2003; Xin and Sutman 2011). As the SOFA-app was co-developed with the autism community, the involvement of those developing and delivering social stories in the design process would be expected to ensure that the technology provided the appropriate level of support for those using it (see Constantin et al. 2017; Fletcher-Watson et al. 2019; Parsons et al. 2019; Smith et al. 2020).

In conclusion, using an RCT design with digital technology to reduce variability in social story interventions has demonstrated significant improvements in maladaptive behaviours in children on the autism spectrum. Whilst numbers are small, the present study suggests that increasing methodological rigour and intervention fidelity can provide consistent evidence for social stories.

## Appendix

### Carol Gray’s Social Story™ Criteria (Gray, 2010)

*Criterion 1: The Social Story Goal.*

Each SS needs one clear goal.

*Criterion 2: Two-Step Discovery.*

Information needs to be gathered in order to identify a topic/focus for the SS and to try to understand the situation from the perspective of the child.

*Criterion 3: Three Parts and a Title.*

Each SS needs a title, introduction, main body and conclusion.

*Criterion 4: FOURmat.*

SS should be tailored to meet the individual needs (e.g. ability, learning style, interest etc.) of the child.

*Criterion 5: Five Factors Define Voice and Vocabulary.*

SS should be written using a positive and patient tone. The information should be literally accurate and accurate in meaning. They can be written using the past, present and/or future tense and must be in the first- or third-person perspective.

*Criterion 6: Six Questions Guide Story Development.*

SS answers relevant’wh ‘ questions that describe context, including where, when, who, what, how and why.

*Criterion 7: Seven is About Sentences.*

SS compris Descriptive Sentences (4 types), as well as optional Coaching Sentences (3 types).

*Criterion 8: A GR-EIGHT Formula.*

Every SS must have more Descriptive Sentences. The following formula should be adhered to: number of descriptive sentences/number of coaching sentences ≥ 2.

*Criterion 9: Nine Makes it Mine: And Refine.*

SS should be tailored to meet the interests and individual needs of the child: Review and revise SS as necessary to ensure it meets all criteria.

*Criterion 10: Ten Guides to Implementation.*

(1) Edit; (2) Plan for Comprehension; (3) Plan Story Support; (4) Plan Story Review; (5) Plan a Positive Introduction; (6) Monitor; (7) Organize the Stories; (8) Mix & Match to Build Concepts; (9) Story Re-runs and Sequels to Tie Past, Present, and Future; (10) Recycle Instruction into Applause.

## Full Text for Social Stories

### ‘Sharing my Toys’

My name is ___.

At school I like to play with toys.

My favourite toys are the trains and cars.

Sometimes children can share toys, this is good.

I can share by letting my friends have a go with the toys I am playing with.

It makes my friends happy when I share.

It is good to share.

It makes ___, ___ and ___ really happy when I share my toys. (insert teachers name).

I will try and remember to share my toys.

### ‘Sitting Together with the Rest of My Class’

Sometimes in class we sit together at the table or on the carpet.

We sit together to do our work.

When we sit together my friends try hard to listen, so they can learn.

It is really important to listen and learn at school.

Sometimes children find it difficult to sit with the rest of the class at the table or on the carpet, as they want to do other things.

They may want to play instead of doing work.

This makes it difficult for the other children to listen and learn.

It is important for everyone to sit together during lessons so everyone can learn.

If I sit at the table or on the carpet with my friends and do my work, I will be able play afterwards.

My teachers will be pleased with me when I sit together with my class and do my work.

I will try and remember to sit with my friends at the table or on the carpet for lessons to do my work.

### ‘I Can Stay Focused and on Task!’

At school it is my job to stay focused and on task during lessons.

Staying on task means keeping focused on the work that I am doing.

It also means paying attention to what the teacher is saying.

Sometimes when I am working, or the teacher is talking, I get distracted.

Sometimes I get distracted by noises or other students.

I may get distracted by my own thoughts and ideas too.

It is important to stay focused on what the teacher is saying. When you listen and stay focused on the teacher, you will learn a lot in school.

After I stay on task for a long time, I feel proud of myself because I have learnt something new, and I know ____ and ____ will be proud of me. (teachers names).

Staying focused and learning new things will help me achieve my dreams of being a ninja!

When I stay on task and focused during lessons I will also get pacing time or iPad time at the end of the day.

So, let’s stay on task and focused in lessons. Let’s try not to daydream. Then we’ll learn a lot in school every day!

### ‘Waiting When I Leave the Classroom’

At school it is important to learn to wait.

Learning to wait means taking turns and being patient.

At school everyone has to wait their turn before they leave the classroom.

Sometimes I want to leave the classroom, such as to go to lunch or to go home at the end of the day, but I have to wait.

Sometimes instead of waiting for their turn to leave the classroom people get too close to their friends and push past them.

It may hurt my friends or make them unhappy if I push past them when leaving the classroom.

When I want to leave the classroom, I need to look and see if anyone else is there.

Instead of getting too close and pushing past my friends who are waiting it is important give my friend’s personal space.

I should also say “Excuse me!” instead of pushing past my friends so they know that I want to get past.

This shows that I care and respect my teachers and friends and makes everyone feel good!

I will try and wait my turn when leaving the classroom instead of pushing past my friends.

### ‘Sharing the Bikes on the Playground’

At school I love to play with the toys on the playground!

My favourite toys on the playground are the bikes!

Sometimes children can share the bikes that are on the playground, this is good.

I can share by taking it in turns with my friends on the bikes.

It makes my friends so happy when I share the bikes on the playground!

Sometimes instead of sharing the bikes, children may snatch the bikes from other children.

This could hurt or upset others.

When I share the bikes on the playground, it makes other children more likely to share the bikes with me, so we can all have lots of fun!

It makes ____ and ___ very happy when I share the bikes on the playground. (teachers names).

It is good to share the bikes with my friends on the playground.

I will try and remember to share the bikes that I am playing with while on the playground.

### ‘I Should Get Attention Like This’

At school some children irritate, tease or wind up their friends to get attention. This is negative attention.

This may annoy others and they may get angry or upset.

If people irritate or wind up their friends they won’t want to hang out or play anymore.

A good friend does not tease or irritate their friends.

If a friend says to you, “Please stop doing that, I don’t like it!” It is important to listen.

Try to listen to your friends. When they ask you to stop doing something, you should stop. Try and do positive things instead. Talk to them, play, or tell jokes instead. Good friends do this!

It is much better to get attention from doing positive things, it makes my teachers and friends really happy!

If I am kind to my friends they will want to play and hang out with me. I will have lots of friends at school! We will all be really happy!

### ‘Personal Space’

At school it is important to give my friends and teachers personal space.

Personal space is the distance that makes people feel comfortable, it is like an invisible bubble that surrounds you and makes you feel safe.

It also means keeping your hands to yourself.

This is ____ in her personal space. (*insert name).*

This is ____ in his personal space. (*insert name).*

This is ____ in his personal space. (*insert name).*

This is me in my personal space.

When people get too close to their friends and don’t keep their hands to themselves, it can make others feel uncomfortable or upset.

People may walk away or not want to play with me if I get too close and touch.

Sometimes I get too close to my friends and touch them because I am happy to see them.

Instead I can give a ‘thumbs up’, a big smile or say “hello!” to show that I am happy to see them.

If I give my friends personal space they will talk to me, they will play with me.

It makes my friends and teachers really happy when I give people personal space and keep my hands to myself.

I will try and remember to sit or stand nicely in my own space. I will try not to touch my friends. Then we will all feel comfortable and happy at school!

### ‘When I Don’t Get My Way’

My name is ____.

At school I love to play with different toys!

I also like to do special jobs in the classroom, because it makes me feel special!

But, at school we don’t always get our own way.

Sometimes, we may not be able to play with the toys we want to play with or we may not get chosen to do certain jobs in the classroom.

Sometimes this makes me feel upset or angry.

When I feel upset, I sometimes cry, shout or make bad choices.

It is okay to feel upset or angry about not getting what I want, but it is better if I stay calm and use my words to tell _____ how I am feeling. (insert teachers name).

There are lots of children in my class and everyone needs a turn doing classroom jobs and playing with the toys!

It makes my friends and teachers really happy when I stay calm when I don’t get my own way.

I will try and remember to stay calm when I don’t get my own way!

### ‘Change is Okay’ by Rachel Hanrahan

My name is ____.

When I go to school most of my days are the same.

I usually have the same lessons at the same times, and playtime and lunchtime are at the same time every day.

But, sometimes there may be a change in my day.

These may only be little changes but they make me feel upset, angry or frustrated.

It is okay to feel like this when there are changes in my day, but if I stay calm during changes it will be easier.

Soon we will be back to our regular schedule and everyday routine.

My day will be fine, little things can change but they are just little things. Then they are over and the day keeps going, I feel fine!

I will try and remember that change is okay!

I will try and stay calm when little things change!

### ‘Sitting Quietly on the Carpet’ by Rachel Hanrahan

Sometimes at school we sit on the carpet to listen to the teacher (insert teacher name).

We sometimes sit on the carpet during lessons or story time.

It is very important to sit quietly and still on the carpet so that all my friends can listen to the teacher.

Sometimes children talk to their friends or move around on the carpet.

This makes it difficult for my friends to listen to the teacher and learn.

It is really important to listen and learn in school.

If I sit still and quietly on the carpet, it will make my friends and teachers very happy!

I will get also get a sticker if I sit quietly on the carpet and I’ll have time afterwards to play with my favourite toys.

I will try and remember to sit quietly and still on the carpet!

## Footnotes

1 Here we use this term, as some members of the autism community have expressed this preference (Kenny et al. 2016).

2 Here we use the term maladaptive behaviour rather than challenging or problem behaviour (e.g. Fulton et al. 2014).

3 https://memory.psych.mun.ca/models/stats/effect_size.shtml.
